# Noble metal-coated MoS_2_ nanofilms with vertically-aligned 2D layers for visible light-driven photocatalytic degradation of emerging water contaminants

**DOI:** 10.1038/s41598-017-14816-9

**Published:** 2017-11-02

**Authors:** Md Ashraful Islam, Jared Church, Changseok Han, Hee-Suk Chung, Eunji Ji, Jong Hun Kim, Nitin Choudhary, Gwan-Hyoung Lee, Woo Hyoung Lee, Yeonwoong Jung

**Affiliations:** 1 NanoScience Technology Center, University of Central Florida, Orlando, Florida 32826 USA; 2 Department of Electrical and Computer Engineering, University of Central Florida, Orlando, Florida 32816 USA; 3 Department of Civil, Environmental, and Construction Engineering, University of Central Florida, Orlando, Florida 32816 USA; 4 Environmental Engineering and Science Program, University of Cincinnati, Cincinnati, Ohio, 45221-0012 USA; 5 Analytical Research Division, Korea Basic Science Institute, Jeonju, 54907 Jeollabuk-do South Korea; 6 Department of Materials Science and Engineering, Yonsei University, Seoul, 03722 Korea; 7 Department of Materials Science and Engineering, University of Central Florida, Orlando, Florida 32826 USA

## Abstract

Two-dimensional molybdenum disulfide (2D MoS_2_) presents extraordinary optical, electrical, and chemical properties which are highly tunable by engineering the orientation of constituent 2D layers. 2D MoS_2_ films with vertically-aligned layers exhibit numerous 2D edge sites which are predicted to offer superior chemical reactivity owing to their enriched dangling bonds. This enhanced chemical reactivity coupled with their tunable band gap energy can render the vertical 2D MoS_2_ unique opportunities for environmental applications that go beyond the conventional applications of horizontal 2D MoS_2_ in electronics/opto-electronics. Herein, we report that MoS_2_ films with vertically-aligned 2D layers exhibit excellent visible light responsive photocatalytic activities for efficiently degrading organic compounds in contaminated water such as harmful algal blooms. We demonstrate the visible light-driven rapid degradation of microcystin-LR, one of the most toxic compounds produced by the algal blooms, and reveal that the degradation efficiency can be significantly improved by incorporating noble metals. This study suggests a high promise of these emerging 2D materials for water treatment, significantly broadening their versatility for a wide range of energy and environmental applications.

## Introduction

Viable solutions for efficiently degrading emerging organic contaminants in drinking water supplies have urgently been demanded with their increasing threats to environment and human health. For instance, harmful algal blooms (HABs), a rapid increase and/or accumulation in the population of algae which can severely damage aquatic ecosystems, have recently gained substantial public attention^1–3^. The primary concern over HABs is that they release harmful cyanotoxins^4,5^, which can be fatal if ingested and/or inhaled^6,7^. However, traditional water purification methods are designed to primarily remove suspended solids and/or individual elements of carbon, nitrogen, and phosphorus in contaminated water, which are not well suited to directly degrade algal toxins. Photocatalysis, an alternative approach based on a solar energy-driven oxidation process, has drawn substantial interest for its intrinsic simplicity and efficient operation^8–11^. In this approach, photoactive catalytic materials in contact with contaminated water generate electron-hole (e^−^h^+^) pairs upon absorbing the solar energy. The photo-generated charge carriers dissociate dissolved oxygen (DO) in water, generating reactive oxygen species (ROS) such as hydroxyl groups and superoxide anions, which in turn disinfect pathogens^12^. Photocatalytic materials (usually, oxide semiconductors) accelerate the rate of the associated chemical reactions (oxidation/reduction) in the microorganisms. However, conventional photocatalytic methods have relied on the use of ultraviolet (UV) light for e^−^h^+^ generation, which is limited to harness a very small portion of the available solar energy^13^. This is because photocatalytic semiconductors possess large band gap (E_g_) energies which match the UV spectrum corresponding to only ~4–5% of the entire solar spectrum, thus, inevitably resulting in prolonged exposure and slow reaction rates. For instance, titanium dioxide (TiO_2_), one of the most sought-after photocatalytic semiconductors presents E_g_ > 3.0 eV^8–11^ and only harvests UV light while neglecting the broad range of the visible light which corresponds to >40% of the entire solar spectrum^14^.

Molybdenum disulphide (MoS_2_), a recently rediscovered semiconductor classified as two-dimensional (2D) transition metal dichalcogenides (TMDs), presents a rich set of optical and structural properties uniquely suitable for photocatalytic reactions. In terms of optical properties; (1) It presents a band gap energy (~1.2–1.8 eV) matching the spectral range of the visible light. Moreover, the band gap energy is highly tunable by varying the number of 2D atomic layers^15^. (2) It exhibits exceptionally large sunlight absorption; for example, over an order of magnitude higher than conventional semiconductors such as silicon (Si) or gallium arsenide (GaAs) for similar thicknesses^16^. In terms of structural advantages; (1) It can be grown vertically standing on growth substrates (e.g. silicon dioxide (SiO_2_)) exposing the edges of individual 2D layers^17–19^. In this vertical orientation, atomically unsaturated 2D edge sites full of molybdenum (Mo) and sulfur (S) dangling bonds are maximally exposed on the surface. Consequently, the surface is highly reactive, offering large chemical/physical adsorption capacity for capturing molecules^20–22^. (2) It presents suitable energy band structure with respect to the redox potentials for hydrogen- or oxygen evolution reactions (HER or OER) as its conduction (valence) band edge lies above (below) the electrostatic potential of H_2_ (O_2_) evolution, respectively^22–26^. A proof-of-concept demonstration of its application to microbial inactivation in contaminated water has recently been reported^12^, which studies the disinfection of *Escherichia (E.) coli* for drinking water purification *via ex-situ* photocatalytic measurements.

In this work, we demonstrate rapid and efficient photocatalytic degradation of algal toxins using MoS_2_ films with vertically-aligned 2D layers under visible light illumination. Particularly, we study the degradation of one of the most toxic organic compounds generated from harmful algal blooms, microcystin-LR (MC-LR), which is recognized as an emerging threat to a wide range of water sources, including seawater, river, and lakes. We investigate its photocatalytic reaction kinetics by *in situ* characterization of ROS generation using microsensors. Moreover, we identify that the coating of thin noble metal layers on top of pristine MoS_2_ films significantly improves the photocatalytic efficiency, enabling the rapid and complete removal of MC-LR. The underlying mechanisms for the observed photocatalytic reactions as well as their governing parameters are also discussed. The study suggests the promise and versatility of MoS_2_ films with vertically-aligned 2D layers for a broad range of water purification and environmental applications.

## Result and Discussion

**Figure 1:**
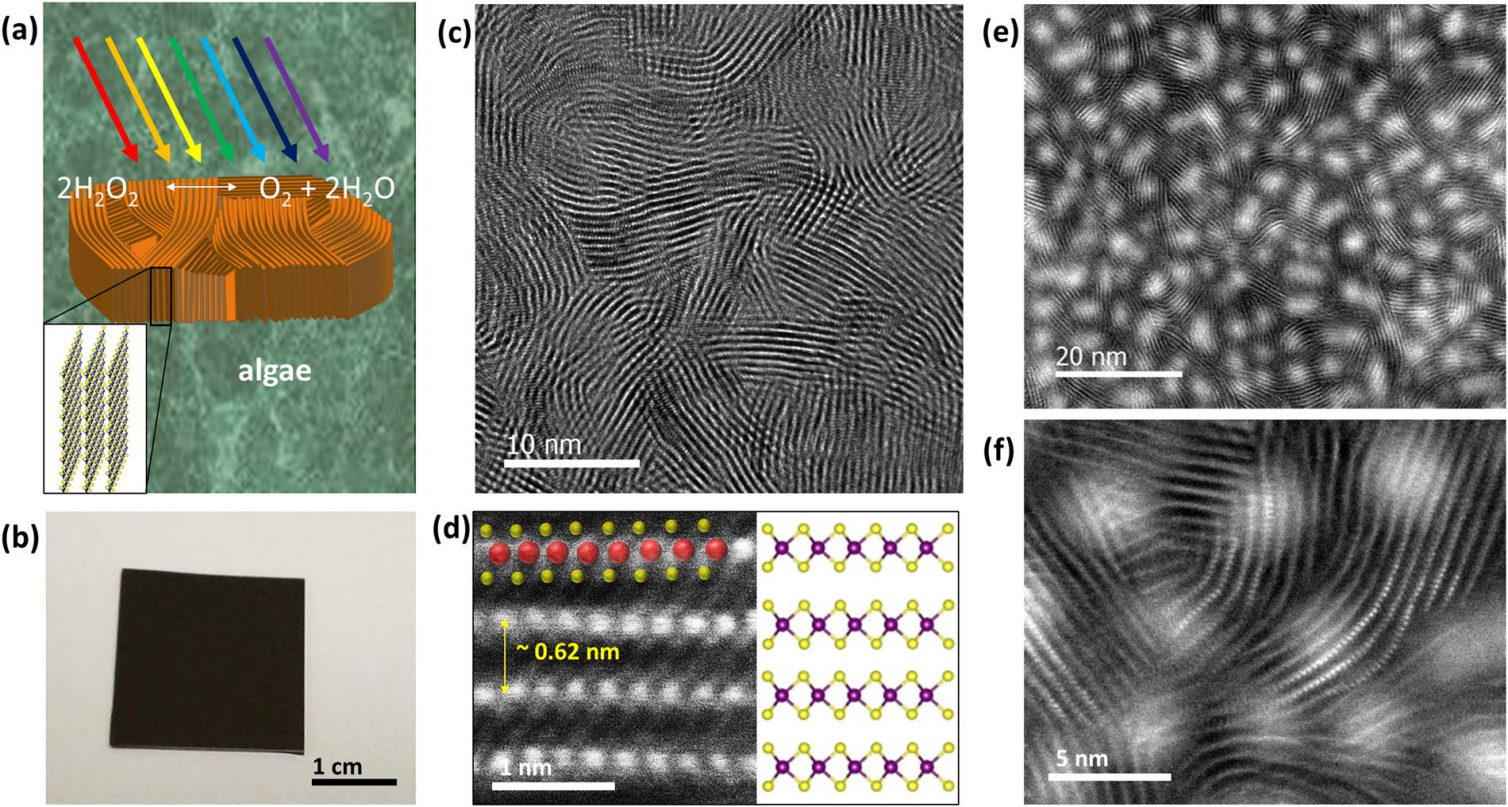
Concept for photocatalytic degradation of MC-LR and TEM characterizations of MoS_2_ films with vertically-aligned layers. **(a)** Schematic illustration for photocatalytic degradation using MoS_2_ films. **(b)** Image of an as-grown MoS_2_ film. **(c)** HRTEM image to show vertically-aligned 2D MoS_2_ layers. **(d)** ADF-STEM image and atomic structure comparison of vertically-aligned 2D layers. **(e)** HRTEM image to show the uniform distribution of Pt nanoparticles on the vertically-aligned 2D layer edges. **(f)** Close-up image to show that vertical 2D layer edges are well maintained even after Pt incorporation.

Figure 1(a) is the schematic illustration demonstrating the concept of photocatalytic degradation of emerging water contaminants using semiconducting 2D MoS_2_ photocatalysts. MoS_2_ films with vertically aligned 2D layers grown on SiO_2_/Si substrates are immersed in a water bath containing algal toxins (MC-LR in this case) absorbing photons from the visible light, which readily generates e^−^h^+^. These charge carriers migrate to the semiconducting surface where they react with hydroxyl ions and oxygen compounds to generate highly reactive species (i.e. O_2_^•−^,.OH, and H_2_O_2_) that can degrade  the algal toxins. Figure 1(b) is an image of a MoS_2_ film with vertically aligned 2D layers grown on a SiO_2_/Si wafer of >2 cm^2^ in size. The growth of the MoS_2_ film with vertically aligned 2D layers was achieved by the sulfurization of Mo-deposited wafers following the previously reported method^17^. Details for the material growth are in *Materials and Methods*. Figure 1(c) is a representative high-resolution transmission electron microscopy (HRTEM) image of a MoS_2_ film with vertically-aligned 2D layers. It is apparent that the MoS_2_ film predominantly exposes the edge sites of vertically-aligned 2D layers perpendicular to the substrate surface. The film presents continuously connected vertically-aligned 2D MoS_2_ layers, uniformly covering the entire surface (Supplementary Information, S1). The annular dark field (ADF) scanning TEM (STEM) image in Fig. 1(d) allows for a near atomic-scale investigation of a single MoS_2_ grain with vertically-aligned 2D layers. The image clearly identifies the individual atomic planes of molybdenum (Mo) and sulfur (S) organized in an S-Mo-S sequence with heavier Mo atoms appearing brighter than S atoms. The projected atomic structure of MoS_2_ is superimposed on the image, indicating an interlayer spacing of ~0.62 nm. The presented structural model matches the image of the projected MoS_2_ atomic structure, indicating that the MoS_2_ film predominantly expose the semiconducting 2H hexagonal phases^27,28^. For the purpose of photocatalytic performance tests, some MoS_2_ films with vertically-aligned 2D layers were coated with thin layers of noble metals. Figure 1(e) shows a HRTEM image of a platinum (Pt; ~3 nm thick)-deposited MoS_2_ film with vertically-aligned 2D layers. The image reveals that individual Pt nanoparticles are uniformly and discontinuously distributed, while the vertical 2D layer edges are well maintained even with the Pt coating (Fig. 1(f)).

**Figure 2:**
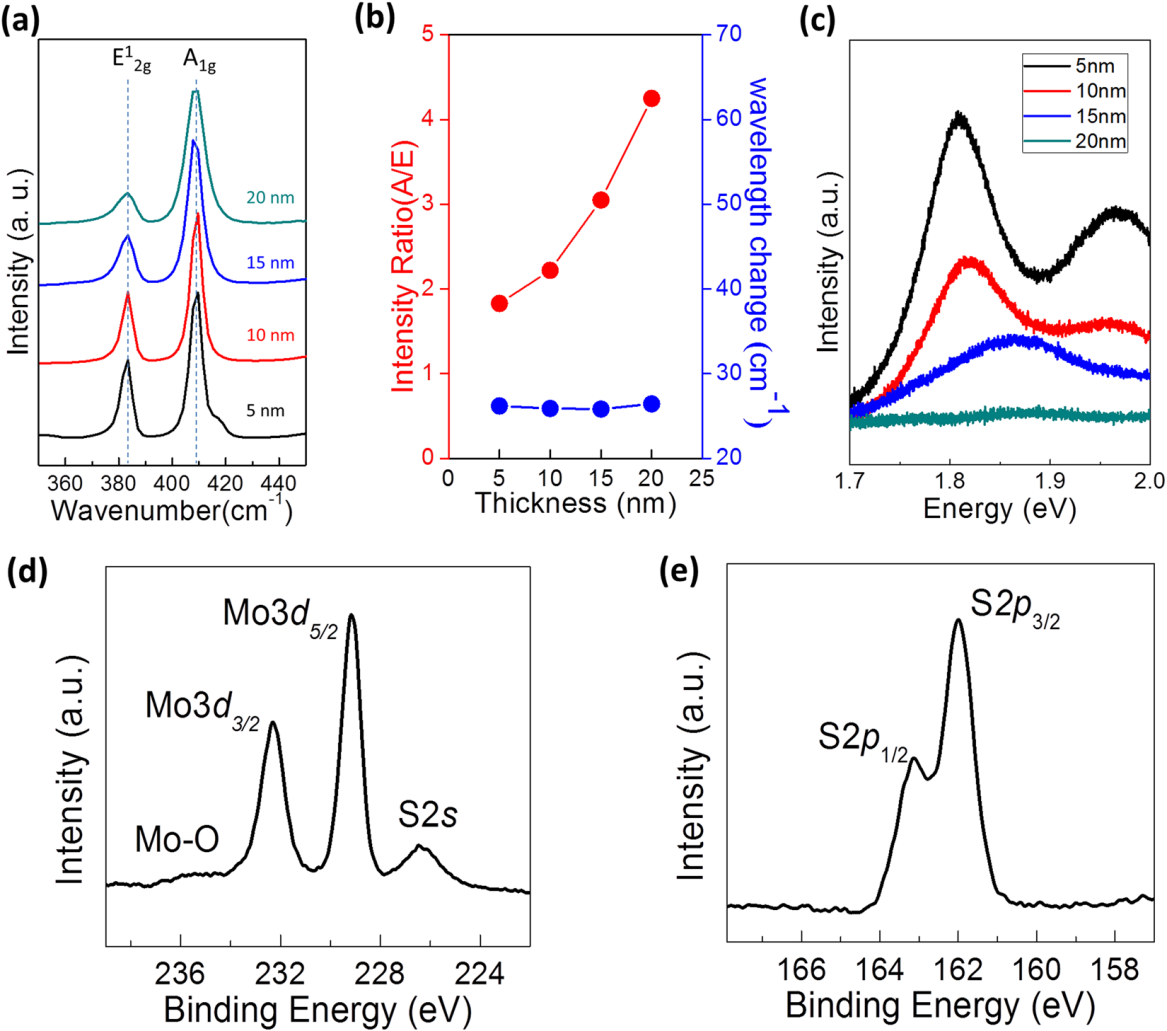
Structural and chemical characterizations by Raman, PL, and XPS. **(a)** Raman spectra obtained from the MoS_2_ films grown with Mo seed layers of various thicknesses. **(b)** A_1g_/E^1^_2g_ intensity ratio and frequency difference of A_1g_−E^1^_2g_ as a function of Mo thickness. **(c)** PL spectra obtained from the corresponding MoS_2_ films. XPS spectra of a MoS_2_ film for **(d)** Mo3*d* and **(e)** S2*p* core levels.

Detailed structural and chemical characterizations were further performed. In Fig. 2(a), Raman spectra obtained from the MoS_2_ films grown with Mo seeds of various thicknesses are presented. For all film thicknesses, the Raman spectra show strong signatures of both the in-plane (E^1^_2g_) and out-of-plane (A_1g_) phonon modes of MoS_2_. The intensity ratio of A_1g_ mode to E^1^_2g_ mode (A_1g_/E_2g_) increases with increasing Mo thickness, which indicates the pronounced exposure of 2D edge sites and is consistent with previous studies^17^. Figure 2(b) shows the change in the A_1g_/E_2g_ intensity ratio as a function of Mo thickness (red), while there is no significant thickness-dependent change in the frequency difference of A_1g_-E^1^_2g_ (blue). The thickness of the MoS_2_ film with vertically-aligned 2D layers was identified by cross-section TEM characterizations (Supplementary Information, S2). The thickness of initial Mo seeds increases by ~3 times after the sulfurization, which was consistently observed for Mo thickness from 5 nm to 20 nm. Figure 2(c) is the photoluminescence (PL) spectra obtained from the same MoS_2_ films with vertically-aligned 2D layers in Fig. 2(a) and (b). We observe that MoS_2_ films with a thickness of <10 nm exhibit two emission peaks centered around 1.81 eV and 1.96 eV, corresponding to the A and B excitons of MoS_2_^27^. The observation of such strong PL peaks is interesting as they are typically observed in mono-to-few layer horizontal 2D MoS_2_ flakes of much smaller thickness (<a few nm)^27^. Similar observations of PL peaks in vertically-aligned 2D MoS_2_ layers have previously been reported^29^, while the exact mechanism for the PL emission remains unclear at present. X-ray photoelectron spectroscopy (XPS) characterizations were performed to investigate the chemical composition and the atomic bonding characteristics of the MoS_2_ film with vertically-aligned 2D layers. Figure 2(d) and (e) show the XPS spectra for the Mo3*d* and S2*p* core levels of the studied sample. The absence of a noticeable peak at 235.2 eV corresponding to Mo-O bonds indicates a negligible formation of molybdenum oxides. No noticeable peaks corresponding to the S-O formation in the S2*p* states are observed, which indicates the negligible oxidation of sulfur in the sample. The chemical compositions of the MoS_2_ film was determined by analyzing the Mo3*d* and S2*p* peak areas with relative sensitivity factors of 9.5 and 1.67, respectively. The atomic ratio of Mo and S is identified to be ~1:1.85, indicating a small density of S vacancies, which is commonly observed chemically synthesized MoS_2_ and is known to introduce n-type intrinsic doping^30^.

**Figure 3:**
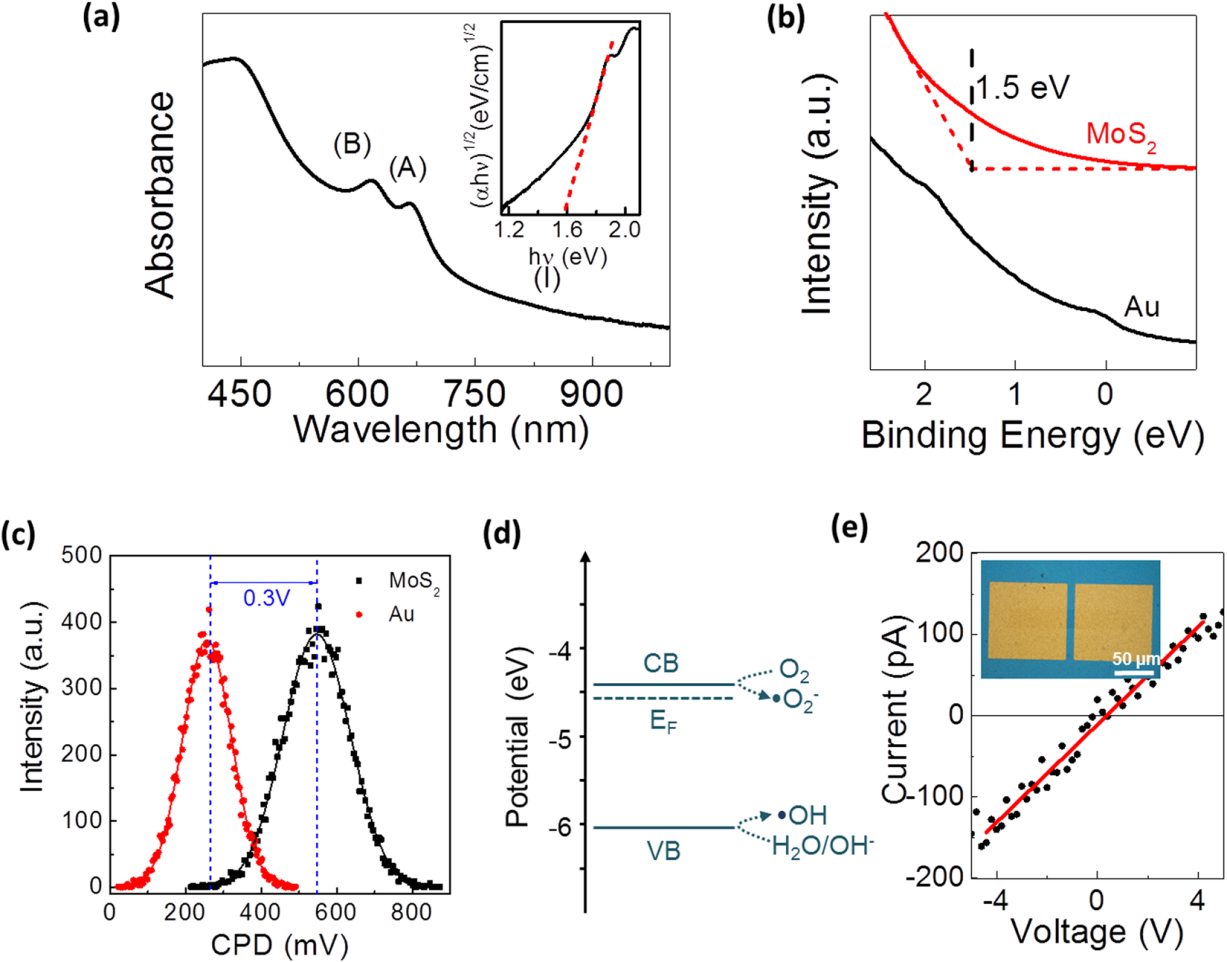
Band structure determination by optical and electrical characterizations. **(a)** UV-vis spectrum from a MoS_2_ film with thickness of 20 nm. The inset shows the extraction of band gap (E_g_). (**b**) UPS measurement for the determination of VB position in the MoS_2_ film. (**c**) Surface potentials of MoS_2_ and reference Au measured by scanning kelvin probe microscopy. (**d**) Band structure of the MoS_2_ film with respect to the redox potentials for hydrogen- or oxygen evolution reactions. (**e**) Current-voltage characteristics of a MoS_2_ film on a SiO_2_/Si substrate. Inset shows the optical image of the corresponding device.

Electronic band structures of the MoS_2_ films with vertically-aligned 2D layers were identified to assess their feasibility for visible light-driven photocatalytic reactions. Figure 3(a) shows the UV-vis spectra obtained from a MoS_2_ film (thickness: ~20 nm) transferred on a transparent sapphire substrate. Two prominent exciton absorption peaks corresponding to A and B excitons at ~1.8 eV and ~2.0 eV are observed, which originates from the strong spin-orbit splitting of the valence band. The absorbance tail observed in the regime I at a wavelength < 1.8 eV indicates the indirect band transition (Fig. 3(a) inset). The band gap (E_g_) of the sample was estimated by using the Tauc’s equation, (α(ν)*hν)^1/n^ = *A*(*hν* − *E*_*g*_), where *α(ν)*, *A*, *n* are the absorption coefficient, proportionality constant, and gap-type depending exponent, respectively^31,32^. In this case, n = 2 is assigned such that multi-layered MoS_2_ presents indirect band gap originating from the maximum of valence band at Γ and the minimum of conduction band halfway between Γ and K^33^. The inset of Fig. 3(a) presents the variations of (*αhν)* ^*1/2*^ *vs. hν* for the MoS_2_ film with vertically-aligned 2D layers. The red straight dashed line indicates the indirectly allowed transition region in the sample, from which E_g_ of ~1.59 eV is extracted. This value of E_g_ belongs to the visible light regime of the solar spectrum, indicating that the MoS_2_ film with vertically-aligned 2D layers can absorb sun light up to ~780 nm. This enhanced absorption corresponds to ~50% increase in energy compared to other oxide semiconductor photocatalysts which are sensitive to UV light only (i.e. 4–5% of the whole solar spectrum). The positions of the valence band (VB) and the work function of the MoS_2_ film were determined by ultraviolet photoemission spectroscopy (UPS) and scanning kelvin probe microscopy (SKPM). The characterizations were performed on the MoS_2_ films transferred onto conductive (e.g. gold (Au)-deposited) Si/SiO_2_ substrates. Details are in *Materials and Methods*. The VB position is identified to be ~−6.07 eV, which is ~1.5 eV lower than the Fermi level of Au as shown in Fig. 3(c). The work function of the MoS_2_ film is extracted to be ~−4.57 eV based on the surface potential difference of ~0.3 V in Fig. 3(c). Figure 3(d) presents the band structure of the MoS_2_ film with respect to ROS reaction potentials. It is noted that the Fermi level of the MoS_2_ film is close to the conduction band (CB) edge implying its n-type carrier transport, as also predicted from XPS analysis which indicates intrinsic n-doping owing to S vacancies. The results indicate that MoS_2_ films with vertically-aligned 2D layers possess electronic structures suitable for ROS generation and photocatalytic reactions^12^. The carrier transport properties of the MoS_2_ film with vertically-aligned 2D layers were also evaluated by measuring its sheet resistance (R_s_). Two metal contacts were deposited on the either sides of the as-grown MoS_2_ film channel defined by e-beam lithography (Fig. 3(e) inset). The current-voltage characteristic in Fig. 3(e) shows Ohmic transport with R_s_ of ~2.63 × ${10}^{9}$ Ω/□. The sheet resistance of the MoS_2_ film is observed to be three orders of magnitude larger than that of monolayer horizontal MoS_2_ flakes where the carrier transport occurs on the basal planes of 2D layers. This large in-plane R_s_ of the vertically-aligned 2D MoS_2_ layers indicates the hopping-dominated carrier transports accompanied by significant carrier scattering across the van der Waals gaps in between vertical 2D layers^34^.

**Figure 4:**
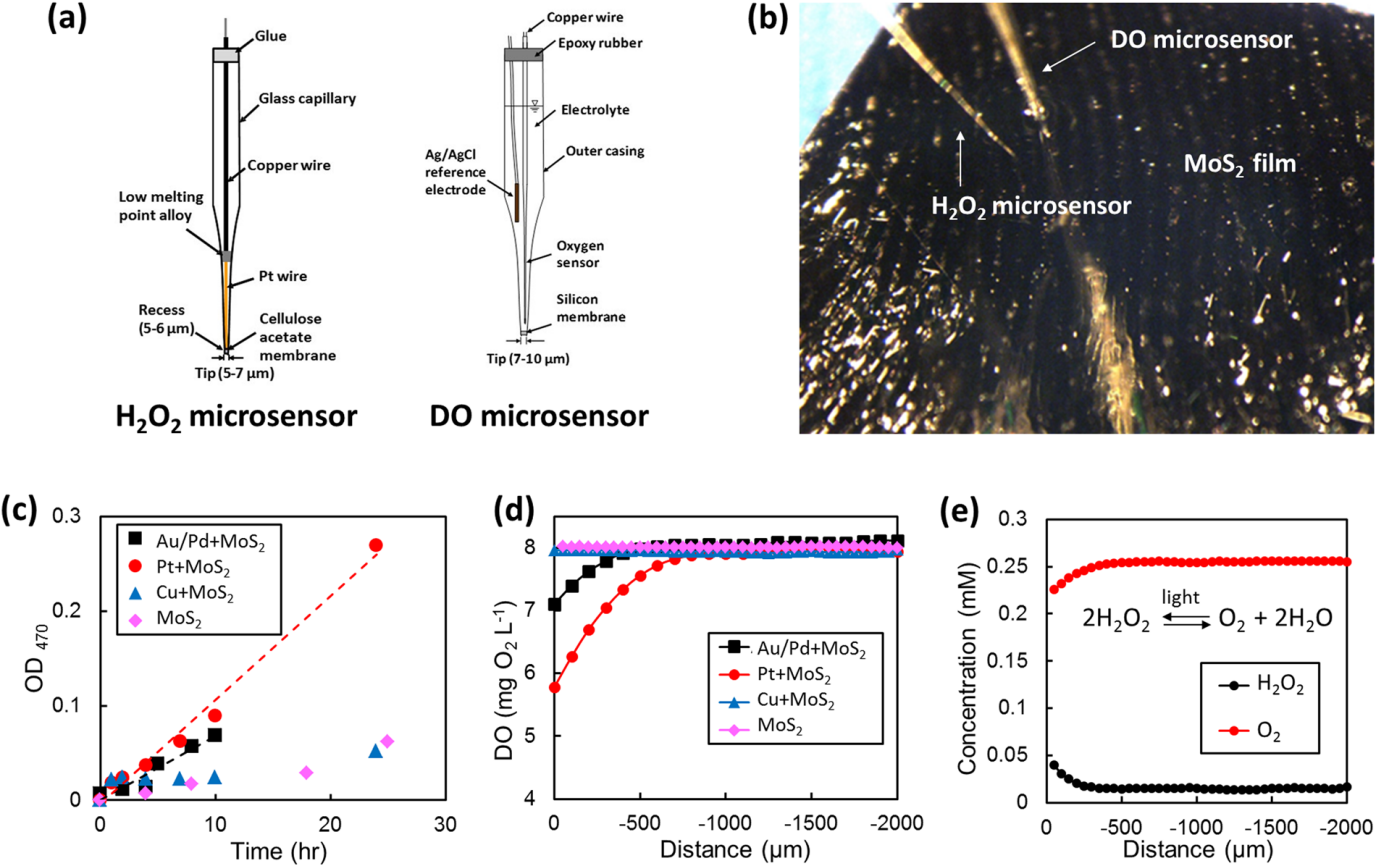
*In situ* monitoring of ROS production. (**a**) H_2_O_2_ and dissolved oxygen (DO) microsensors for *in situ* characterizations of H_2_O_2_ production and O_2_ consumption **(b)** Image of H_2_O_2_ and DO microsensors immersed in a water bath **(c)** ROS production from MoS_2_ films of various types measured by absorbance of XTT-formazan at 470 nm (optical density [OD] _470_). (**d**) DO concentration microprofiles of various MoS_2_ films **(e)** H_2_O_2_ and DO concentration microprofiles at the surface of Pt-coated MoS_2_ film. 0 µm represents the top surface of the film. All the microprofiles were obtained after ~30 min exposure to water.

Photocatalytic performances of various MoS_2_ films were evaluated for the visible light-driven production of ROS and degradation of MC-LR. The tested samples include pristine MoS_2_ and MoS_2_ films coated with thin (5 nm) noble metals (platinum (Pt), copper (Cu), gold/palladium (Au/Pd)). The production of ROS was monitored using H_2_O_2_ detection microsensors and 2,3-bis (2-methoxy-4-nitro-5-sulfophenyl)-2H-tetrazolium-5-carboxanilide inner salt (XTT) essay. In photocatalysis, chemical reactions occur at the photo-reactive surfaces in aqueous solutions, thus *in situ* measurements of the mass transfer of reactants and products are essential to understand the overall photocatalytic reactions^35^. The concentration gradient microprofiles of O_2_ and H_2_O_2,_ an indication of ROS production, were directly measured using a commercial DO microsensor and a home-built Pt-based H_2_O_2_ microsensor (Fig. 4(a)). For *in situ* microprofiling, MoS_2_ samples were placed inside a microprofile chamber^36^ with a 2 mLmin^−1^ continuous flow of deionized (DI) water and a silver/silver chloride (Ag/AgCl) was used as a reference electrode (Fig. 4(b)). The ROS production from various MoS_2_ films immersed in MC-LR baths was quantified by the optical absorption of XTT at 470 nm wavelength. An increase in the optical density (OD) indicates an increase of ROS concentration (i.e. O_2_^•−^) under illumination^37^. Figure 4(c) shows the ROS production (denoted as OD_470_) from MoS_2_ films of various types as a function of illumination time. It is clear that all the tested films present noticeable ROS production, exhibiting a linear relationship in OD_470_ *vs*. time. Amongst them, the Pt-coated MoS_2_ film exhibits the highest ROS production rate (determined from the slopes of the plots). DO concentration microprofiles of the corresponding samples are shown in Fig. 4(d), which indicates O_2_ concentrations in solutions as a function of the distance from the photo-reactive surface. The Pt-coated MoS_2_ exhibits the largest consumption of oxygen with a surface concentration of 5.8 mg O_2_ L^−1^, which corresponds to 2.2 mg O_2_ L^−1^ consumption in comparison to the bulk concentration (8.02 ± 0.2 mg O_2_ L^−1^). The result suggests that Pt-coated MoS_2_ films are highly photocatalytic under visible light illumination, and are consistent with the XTT characterizations (Fig. 4(c)). The detailed kinetics of ROS production in Pt-coated MoS_2_ films were further revealed by quantifying the conversion ratio of O_2_ to H_2_O_2_ using the H_2_O_2_ and DO microsensors (Fig. 4(e)). Details for the microsensor characterizations are in *Materials and Methods*. The microprofiles reveal that 60% of the O_2_ consumed at the film surface is being stoichiometrically converted to H_2_O_2_, following 2H_2_O_2_ ↔ O_2_ + 2H_2_O. The overall results obtained from XTT analysis and *in situ* microsensor characterizations confirm that Pt-coated MoS_2_ films efficiently produce ROS under visible light.

**Figure 5:**
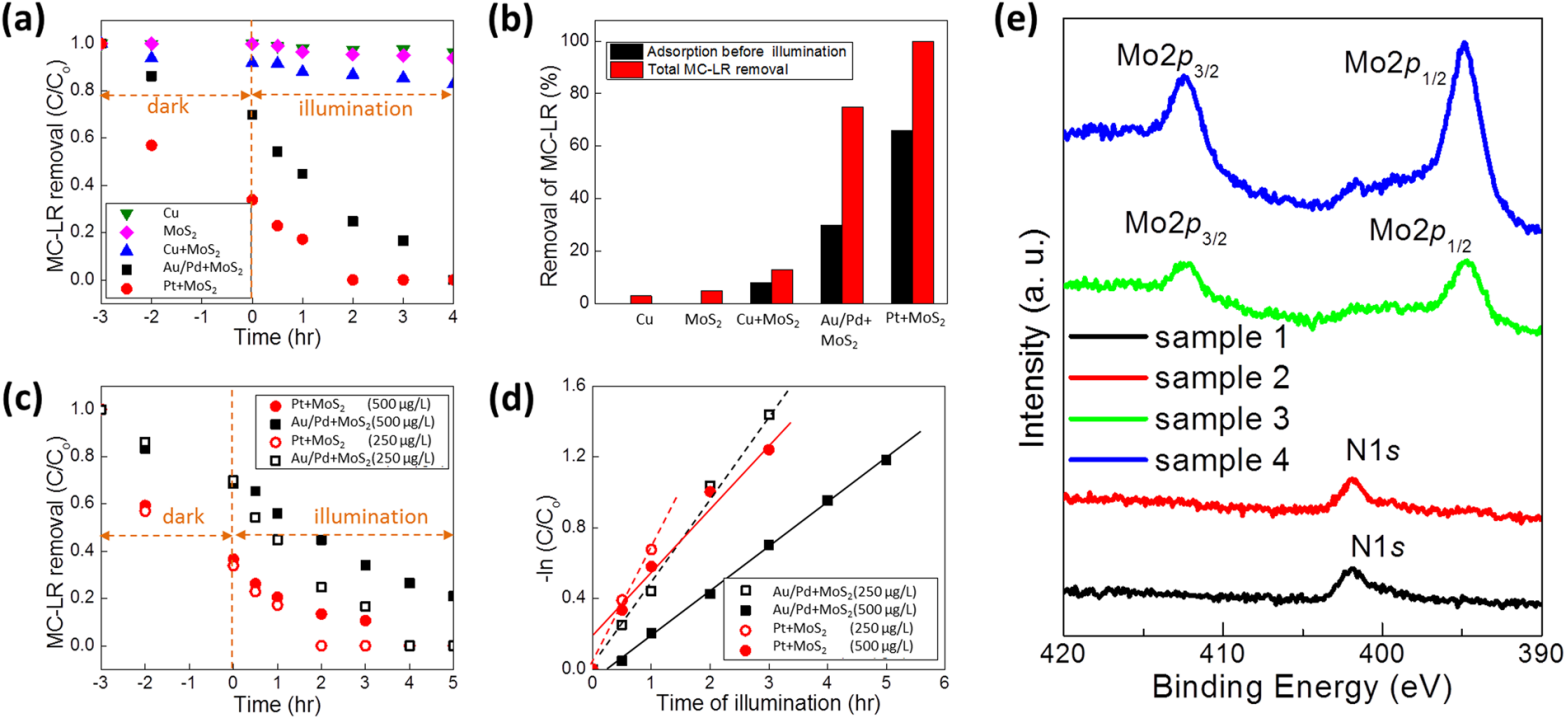
Photocatalytic degradation of MC-LR at pH 5.8. **(a)** Removal of MC-LR as a function of illumination time (initial MC-LR concentration of 250 μg L^−1^). **(b)** Comparison of adsorption with various samples after two hour illumination. **(c)** Comparison of MC-LR removal for Pt + MoS_2_ and Au/Pd + MoS_2_ at concentrations of 250 μg L^−1^ and 500 μg L^−1^. **(d)** Determination of rate constants for the photocatalytic degradation of MC-LR with Pt + MoS_2_ and Au/Pd + MoS_2_ based on the pseudo first-order kinetics. (**e**) XPS characterizations to show the appearance of N1s peaks in MC-LR tested samples (sample 1 and 2) in comparison to pristine samples (sample 3 and 4). Sample 1,3 and 2,4 were prepared from Mo films of 10 nm and 15 nm thickness, respectively.

The direct photocatalytic removal of MC-LR with various MoS_2_-based films using a visible light illuminating fluorescent lamp (Spectrum data is in Supplementary Information, S3) was further investigated in Fig. 5. Figure 5(a) presents the relative concentration (normalized to initial concentration) of removed MC-LR as a function of time. Pt-coated MoS_2_ films present the fastest removal of MC-LR under identical conditions (pH = 5.8 and initial MC-LR concentration = 250 µg L^−1^), achieving a complete removal within 2 hours after the onset of illumination. It is worth mentioning that pristine MoS_2_ films also remove MC-LR slightly faster than Cu-only film, indicating the intrinsic photocatalytic activity of vertical MoS_2_ with E_g_ tailored to visible light. Cu-coated MoS_2_ films exhibit higher removal rate than both pristine MoS_2_ and Cu-only, indicating that metal coatings noticeably improve the overall degradation efficiency. Moreover, it is interesting to note that the removal of MC-LR occurs even before illumination (in the dark), which is attributed to the adsorption of MC-LR to the samples. Figure 5(b) compares the contribution of adsorption and photocatalytic activity that account for the total degradation of MC-LR. The plots reveal that pristine MoS_2_ films do not exhibit a noticeable removal of MC-LR by adsorption-only, further indicating their intrinsic photocatalytic activity. Thus, the significantly improved removal efficiency achieved in Pt-coated MoS_2_ films is likely the result of the synergetic effects of adsorption (mainly contributed by Pt) and photocatalytic reaction (contributed by both Pt and MoS_2_).

Additional experiments were performed using Pt- and Au/Pd-coated MoS_2_ samples using MC-LR with initial concentration of 500 µg L^−1^ and were compared to the results obtained with 250 µg L^−1^ (Fig. 5(a)). Figure 5(c) shows that both samples efficiently degrade MC-LR under illumination while the degradation rate decreases with increasing MC-LR concentrations. The detailed kinetics for the degradation of MC-LR were analyzed. Figure 5(d) presents the degradation kinetics for both Pt- and Au/Pd-coated MoS_2_ in the logarithmic plots of MC-LR concentration as a function of illumination time. The plots indicate that MC-LR degradation follows pseudo first-order kinetics, yielding rate constants of 0.2451 and 0.4884 h^−1^ for Au/Pd and 0.4084 and 0.6769 h^−1^ for Pt, respectively, obtained from 500 µg L^−1^ and 250 µg L^−1^ of MC-LR. These results indicate that the incorporation of noble metals into photocatalytic MoS_2_ films significantly improve the efficiency of e^−^h^+^ separation resulting in enhanced photocatalytic activity. Similar results have previously been reported with other photocatalytic semiconductors which possess much larger E_g_, thus, are sensitive to UV light-only^38–41^. In order to clarify the adsorption-driven removal of MC-LR in the dark, we performed additional XPS characterizations (Fig. 5(e)). We focused on comparing the characteristics of N1s signals for MoS_2_ samples exposed to MC-LR in the dark and pristine MoS_2_. Figure 5(e) shows the XPS spectra from the MC-LR tested (sample 1 and 2) and pristine (sample 3 and 4) samples for the binding energies between 390 eV–420 eV. The results reveal that the MC-LR tested samples present pronounced N1s peaks which are absent in pristine MoS_2_ films, indicating the significant adsorption of MC-LR in the dark. We further investigated the exclusive contribution of MC-LR adsorption in the dark on its overall degradation (Fig. 5(b)) by fitting a type II pseudo second order adsorption model to the MC-LR degradation kinetics obtained under dark/illumination conditions (Supplementary Information, S4). The analysis successfully decouples the adsorption and photocatalytic effects, and further verifies that the observed MC-LR degradation under illumination is a combine result of both the effects.

The effect of noble metals on enhancing photocatalytic activity can be understood considering the following factors; (a) Noble metals of high work functions in contact with n-type MoS_2_ form low-barrier Schottky junctions which facilitate the separation of e^−^h^+^, promoting their migration and participation in ROS generation^42–45^. We identify that our n-type MoS_2_ with vertically-aligned layers (Fig. 3(d)) in contact with Pt form low-barrier Schottky junctions with the Fermi level pinning of MoS_2_ close to its conduction band (Schematic in Supplementary Information, S5). Significantly enhanced photocatalytic activities have also been reported in oxide semiconductor photocatalysts of large E_g_ after incorporating metals^39^, consistent with our studies. (b) Nanoscale noble metals introduce localized surface plasmon resonances (SPR) which significantly increases optical absorption in the visible light spectrum^46^, leading to further enhancement in photocatalytic activity. (c) 2D MoS_2_ with vertically-aligned layers present ~five orders of magnitude greater chemical/physical absorbance compared to horizontal 2D MoS_2_ layers owing to their highly reactive 2D edge sites^47^. It is anticipated that an optimal mass loading of noble metals exists for optimized photocatalytic reactions^39,40,48^, which is to be determined by a balance between enhanced optical absorption and increased electrical conductivity (e^−^h^+^ separation efficiency).

## Conclusion

In summary, we report the visible-light driven photocatalytic degradation of MC-LR using 2D MoS_2_ films with vertically-aligned layers. We reveal that coating thin noble metal layers on top of pristine MoS_2_ films significantly improves the degradation efficiency, resulting in a rapid ROS production and consequent MC-LR removal. The efficient degradation of MC-LR in metal-coated MoS_2_ is attributed to the combined result of the intrinsic photocatalytic activity of MoS_2_ with band gap energy tailored to visible light and enhanced adsorption enabled by noble metals. This study suggests the high potential of 2D MoS_2_ films with vertically-aligned layers for photocatalysts, thus have great implications for a wide range of environmental applications for sustainable emerging contaminants degradation and water purification.

## Materials and Methods

### Synthesis of MoS_2_ films with vertically aligned layers

MoS_2_ films with vertically aligned layers were grown *via* the sulfurization of Mo-deposited SiO_2_/Si substrates in a chemical vapor deposition (CVD) furnace. High-quality Mo films were deposited on Si/SiO_2_ wafers using an e-beam evaporation system (Thermionics VE-100) with the deposition rate of 0.15 Å/s. The Mo/Si/SiO_2_ substrates were placed at the center of a CVD furnace (Lindberg/Blue M Mini-Mite) while an alumina boat containing S powder is located at the upstream side. Following the evacuation down to the base pressure of ~1 mTorr and Ar purging, the CVD furnace was heated to the reaction temperature 650 °C in 15 min and was held for 10 minute under the continuous supply of Ar gas (100 SCCM). After the reaction, the furnace was naturally cooled down to room temperature. The deposition of various metals on MoS_2_ films with vertically-aligned layers was achieved *via* e-beam evaporation with the deposition rate of 0.15 Å/s.

### Structural, optical, and electrical characterizations. Transmission electron microscopy (TEM) characterization

The crystalline structure and the chemical composition of 2D MoS_2_ films with vertically-aligned layers were characterized using a JEOL ARM200F FEG-TEM/STEM with a Cs-corrector. TEM samples were prepared by transferring the 2D MoS_2_ films from SiO_2_/Si substrates to holey carbon TEM grids by using diluted hydrogen fluoride which etches away the SiO_2_. All TEM/ADF-STEM operations were performed at an accelerating voltage of 200 kV.

### Raman and Photoluminescence (PL) characterizations

Raman and PL characterizations were performed with a Raman spectroscopy (Renishaw) with a diode-pumped solid state laser of 532 nm wavelength and a spot size of 1 µm. Raman shifts and PL peaks were obtained under illumination for 10 seconds with power of 156 µW and 2 mW, respectively.

### X-ray photoelectron spectroscopy (XPS) and ultraviolet photoemission spectroscopy (UPS) characterizations

XPS measurements were performed using PHI-5700 spectrometer with monochromatic Al Kα x-ray (1486.6 eV) below 4 × 10^−9^ Torr. The Shirley-type background was removed from the measured XPS spectra. For UPS and absorbance measurements, as-prepared MoS_2_ films on SiO_2_/Si growth substrates were first spin-coated with polymethyl methacrylate (PMMA). The samples were subsequently transferred onto transparent sapphire substrates followed by the etching of SiO_2_ and removal of PMMA.

### UV-Vis absorption and electron transport characterizations

The UV-Vis absorption spectra were obtained using a UV-Vis spectrophotometer (Cary5000, Agilent). The absorption coefficient and the optical energy gap have been determined by characterizing the transmission T(λ) and reflection R(λ) spectra of the samples in the spectral wavelength range of 170~3300 nm. For electrical characterizations, the metal contacts of aluminum/chrome/gold (Al/Cr/Au) (20/5/20 nm) electrodes are fabricated on top of as-prepared MoS_2_ films on SiO_2_/Si substrates using e-beam lithography. Electrical transport properties were measured by a Keithley 4200 semiconductor parameter analyzer.

### Photocatalytic degradation of MC-LR using microsensors

Dissolved oxygen (DO) and hydrogen peroxide (H_2_O_2_) concentration microprofiles were measured using a commercial DO microsensor (10 µm tip size, UNISENSE A/S, Denmark) and a platinum (Pt)-based H_2_O_2_ microsensor fabricated with a 50 µm tip diameter. A 3% hydrogen peroxide solution (H324-500, Fisher) was used to calibrate the H_2_O_2_ microsensor. The DO microsensor was calibrated in oxygen saturated (21% DO, 8.6 mg O_2_ L^−1^ at 23 °C) and deionized (DI) water under nitrogen bubbling (0% DO). The microsensors were calibrated before and after each measurement. For microprofiling, MoS_2_ samples were placed in a microprofile chamber with a 2 mL min^−1^ continuous flow of DI water. Positioning and movement of the microsensor tip towards the sample was accomplished using a three-dimension (3D) micromanipulator (UNISENSE A/S, Denmark) and observed using a stereomicroscope with a CCD camera (World Precision Instruments, Sarasota, FL, USA)^16^. A silver/silver chloride (Ag/AgCl) reference electrode (MI-401, Microelectrodes Inc., Bedford, NH, USA) was positioned using a helping hand (VTHH, Veleman Inc., Forth Worth, TX, USA) and a lab jack (Swiss Boy Model 110, Fisher Scientific) was used to position the MoS_2_ samples in view of the microscope. Microsensor electrical signal (mV or pA) was measured using a multimeter (UNISENSE A/S, Denmark) and the experiments were performed in a Faraday cage (81-334-04, Technical Manufacturing Co. Peabody, MA) to minimize electrical interference. Microprofile measurements were conducted at 50–100 µm intervals with 5 seconds of wait time between each measurement. Three replicate profiles were obtained for each parameter and the microprofiles shown in Fig. 4 are the averaged values of these replicates. Microprofiles were taken from 2,000 µm above the MoS_2_ film surface to the solution surface.

### XTT reduction assay for monitoring ROS production

2-methoxy-4-nitro-5-sulfophenyl-2H-tetrazolium-5-carboxanilide inner salt (XTT) assay (X4626, Sigma Aldrich, St. Louis, MO) was used to investigate ROS production. XTT reduced by superoxide radical anions (O_2_^•−^) generates water-soluble XTT-formazan with a maximum absorption at 470 nm, and the formazan produced by the reaction can be used to determine the relative amount of produced ROS^49^. Metal-coated MoS_2_ films of identical size (3 cm^2^) were tested. 40 mL of XTT (0.4 mM) dissolved in phosphate buffered saline was used to submerge the samples while being exposed to 16,000 Lux (4.47 × 10^−3^ W cm^−2^) continuous cool-white fluorescent light illumination. A shaker table was used at 90 RPM to keep the samples well mixed. Absorbance at 470 nm was taken using a spectrophotometer (DR 900, HACH Co., Loveland, CO) for two days to determine the rate of ROS generation.

### Degradation of MC-LR

The photocatalytic removal of MC-LR in pristine MoS_2_ and metal-coated MoS_2_ films was investigated under an illumination with a fluorescent lamp. As reactors, PYREX™ reusable borosilicate petri-dishes (diameter: 60 mm and depth: 15 mm, Fisher Scientific) were used to contain MC-LR solutions (total volume of 10 mL in each reactor). A stack solution of MC-LR (475815, Calbiochem) of 500 mg L^−1^ was first prepared using SQ water (18 mega ohm of a resistivity) and then diluted to 250 μg L^−1^ or 500 μg L^−1^ with pH = 5.8. Subsequently, MoS_2_ samples were loaded in the reactors covered with aluminum foils and were kept for 3 hours to reach the adsorption equilibrium before illumination (light intensity of 4.75 × 10^−4^ W cm^−2^ measured with Newport broadband radiant power meter (Newport Corporation)). After illumination, the concentration of the MC-LR taken out of the reactors was determined by Agilent series 1100 high-performance liquid chromate graph (HPLC) with a C18 reversed-phase column (Supelco C18 Discover HS column, 150 mm × 2 mm, 5 μm particle size, Supelco, USA). Liquid with a unit volume of 20 μL was constantly injected under the flow rate of 0.2 mL min^−2^, following the previously reported method^50–52^.

## Electronic supplementary material

Supplementary Information
