# Supplementary material for: Noble metal-coated MoS2 nanofilms with vertically-aligned 2D layers for visible light-driven photocatalytic degradation of emerging water contaminants
Source: Sci Rep. 2017 Nov 2;7:14944. doi: 10.1038/s41598-017-14816-9 (PMC5668436; doi:10.1038/s41598-017-14816-9)
Supplement: Supplementary file 1 — Supplementary Information [file 41598_2017_14816_MOESM1_ESM.pdf]

Supplementary Information

Noble metal-coated MoS_2 nanofilms with vertically-aligned 2D layers for visible light-driven photocatalytic degradation of emerging water contaminants

Md Ashraful Islam[+,1,2] Jared Church,[+,3] Changseok Han,[4] Hee-Suk Chung,[5] Eunji Ji,[6] Jong Hun Kim,[6] Nitin Choudhary,[1] Gwan-Hyoung Lee,[6] Woo Hyoung Lee,[3,] Yeonwoong Jung[1,2,7,*]*

1 NanoScience Technology Center, University of Central Florida, Orlando, Florida 32826, USA

2 Department of Electrical and Computer Engineering, University of Central Florida, Orlando, Florida 32816, USA

3 Department of Civil, Environmental, and Construction Engineering, University of Central Florida, Orlando, Florida 32816, USA

4 Environmental Engineering and Science Program, University of Cincinnati, Cincinnati, Ohio 45221-0012, USA

5 Analytical Research Division, Korea Basic Science Institute, Jeonju 54907, Jeollabuk-do, South Korea

6 Department of Material Science and Engineering, Yonsei University, Seoul, 03722, Korea

7 Department of Materials Science and Engineering, University of Central Florida, Orlando, Florida 32826

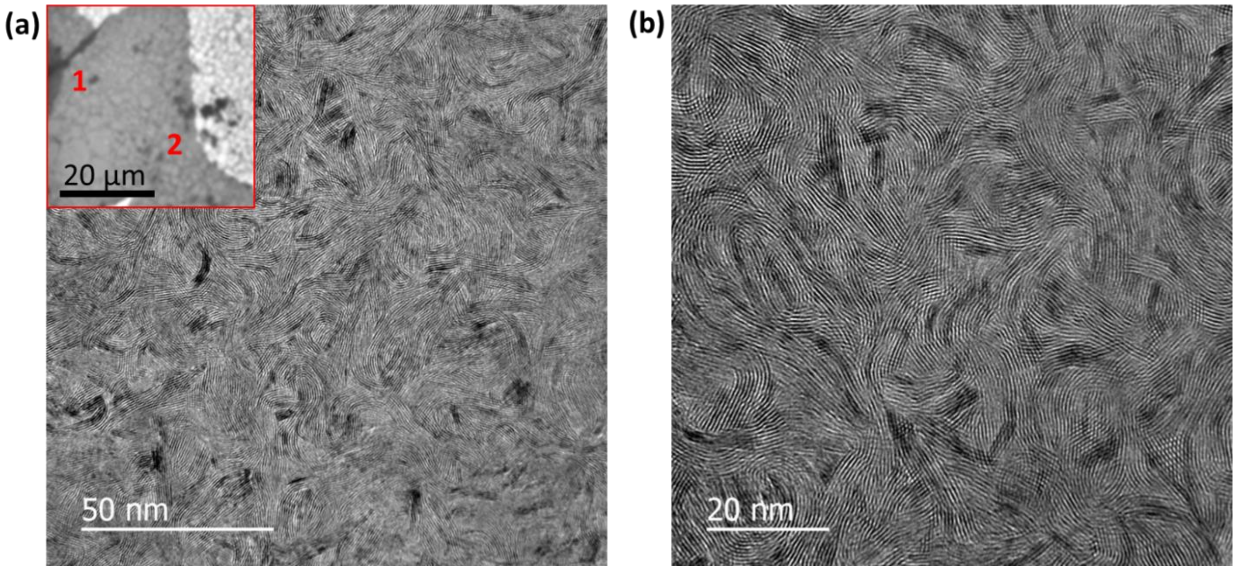

S1. Large-area continuous film ((a) inset) with vertically-aligned 2D MoS_2 layers. (a) and (b) are obtained from 1 and 2 in the inset, respectively.

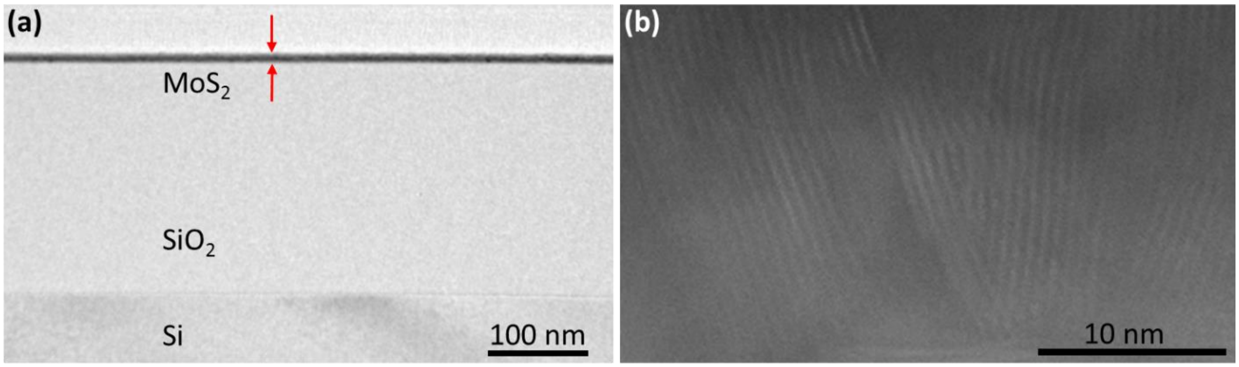

S2. (a) TEM image of a cross-sectioned MoS_2 film grown on a SiO_2/Si substrate. (b) corresponding HRTEM image

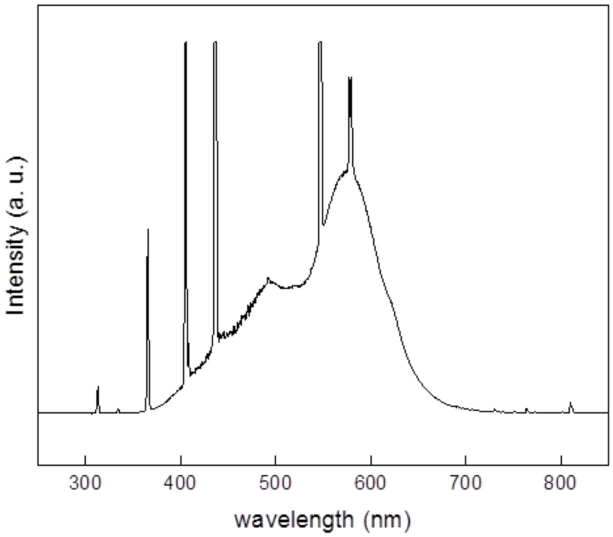

S3. Spectrum profile showing visible light illumination in the wavelength range of ~400 nm to 700 nm.

S4. Verification of dark adsoprtion

Adsorption modeling

Several well-known adsorption models (i.e. pseudo first order, pseudo second order, Elovich, Avrami and fractional power models) were fit to the experimental data and validated using Chi square and normalized standard deviation. The Type II pseudo second order adsorption model best represented our data (X^2=2.45*10^{-6}, R^2=1). This model is the most commonly used mathematical expression for the kinetic data monitored in solid/solution sorption systems [1-3]. The nonlinear form of pseudo second order adsorption is shown in the following equation:

$$q(t) = \frac{k_2 {q_e}^2 t}{1 + k_2 q_e t}$$

Where $q(t)$ and q_e are the total adsorbed amounts of MC-LR per surface area of sorbant at time *t* and equilibrium, respectively. K_2 and q_w were determined by plotting 1/q(t) vs. 1/t. Pt-MoS_2 catalysts displayed faster kinetics compared to Au/Pd-MoS_2 catalysts (K_2=0.47 vs. K_2=0.57 μg cm^{-1} hr^{-1}) and had higher absorption capacity (q_e=0.871 μg cm^{-1} vs. q_e=1.40 μg cm^{-1}). The figure below shows that once the samples are illuminated the MC-LR kinetics increase and exceed the adsorption equilibrium predicted by the dark adsorption model. This finding verifies that the MC-LR degradation is a combined result of both adsorption and photocatalytic reactions. As we clarify below that there is no noticeable production of ROS in dark which is to come from the photo-excited e-h pair generation, this analysis evidences the insignificant “dark” removal of MC-LR driven by photocatalytic reactions, which, in turn, strongly supports the adsorption-driven degradation in dark.

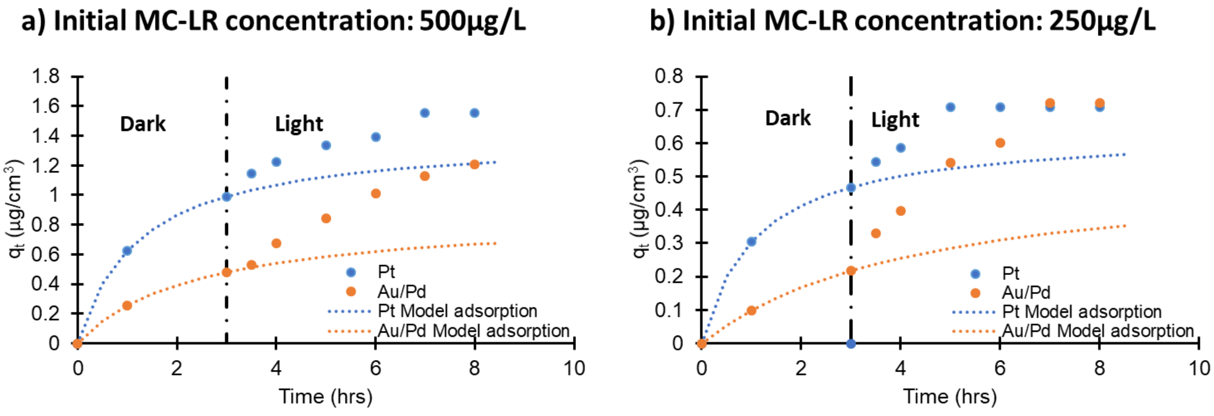

ROS monitoring using XTT

To monitor ROS production by MoS_2, the possibility of superoxide radical anion ($O2^{\bullet-}$) production from MoS_2 was monitored using XTT (2, 3-bis (2-methoxy-4-nitro-5-sulfophenyl)-2H-tetrazolium-5-carboxanilide, Fluka). XTT can be reduced by superoxide radical anions ($O2^{\bullet-}$) to generate water-soluble XTT-formazan with the maximum absorption at 470 nm [4]. Thus, an

increase in absorption at 470 nm indicates an increase of ROS concentration. The below figure shows a preliminary data of ROS production, indicating that Au/Pd coated-MoS_2 with vertically aligned layers shows a higher production rate compared to MoS_2-only within a time frame of 4 hours. Before photoreaction tests, it was confirmed that there is no ROS production under dark using XTT. The Au/Pd coated-MoS_2 sample was colorless throughout the control check in dark which is the same as MoS_2-only and Si-only as shown in the figure.

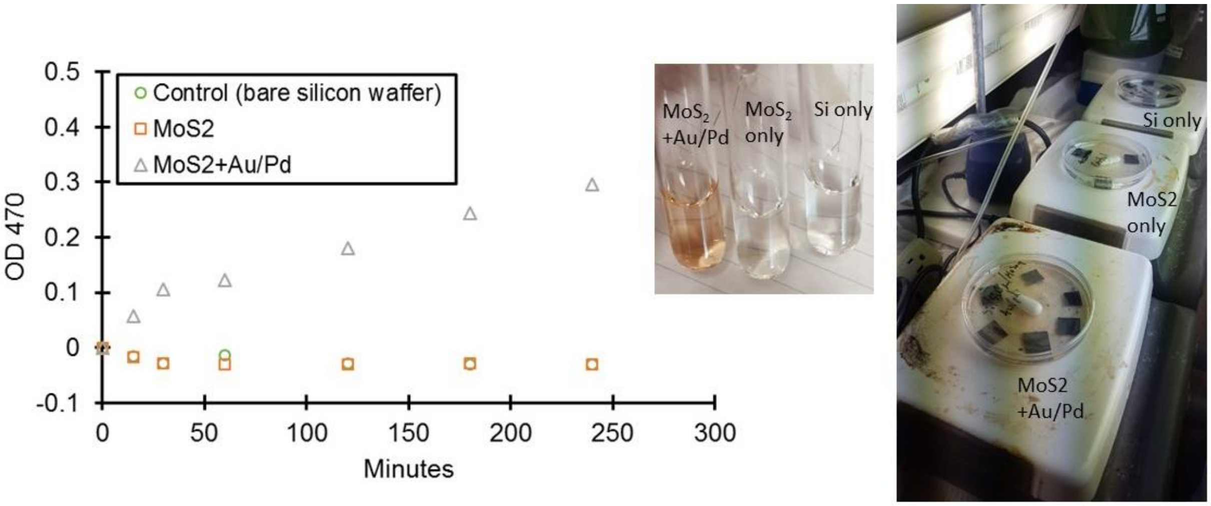

S4. Absorbance of XTT-formazan at 470 nm as a method to monitor the production of ROS from MoS_2, Au/Pd-MoS_2, and a substrate only (silicon wafer without MoS_2 as a control). The surface area of each nanofilm was identical to be 3 cm^2. 40 mL of XTT (0.4 mM) dissolved in phosphate buffered saline was used to submerge the samples while being exposed to 16,000 lx continuous cool-white fluorescent light illumination. Before the tests, it was confirmed that there is no ROS production under dark condition using XTT.

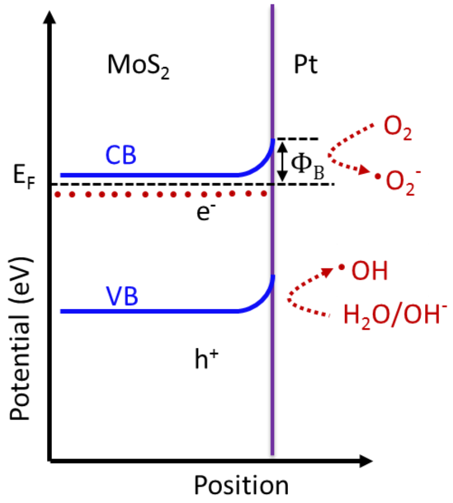

S5. Band structure to illustrate the low-barrier Schottky junction formed by depositing Pt on top of n-type MoS_2

References for Supporting Information, S4

1. Ho, Y.-S.; McKay, G., Pseudo-second order model for sorption processes. *Process Biochem.* **1999,** *34*, (5), 451-465.

2. Gupta, V.; Gupta, B.; Rastogi, A.; Agarwal, S.; Nayak, A., A comparative investigation on adsorption performances of mesoporous activated carbon prepared from waste rubber tire and activated carbon for a hazardous azo dye—Acid Blue 113. *J. Hazard. Mater.* **2011,** *186*, (1), 891-901.

3. Plazinski, W.; Rudzinski, W.; Plazinska, A., Theoretical models of sorption kinetics including a surface reaction mechanism: a review. *Adv. Colloid Interface Sci.* **2009,** *152*, (1), 2-13.

4. Yang, X.; Li, J.; Liang, T.; Ma, C.; Zhang, Y.; Chen, H.; Hanagata, N.; Su, H.; Xu, M., Antibacterial activity of two-dimensional MoS 2 sheets. *Nanoscale* **2014,** *6*, (17), 10126-10133.
